# Preventing Violence by Teachers in Primary Schools: Study Protocol for a Cluster Randomized Controlled Trial in Haiti

**DOI:** 10.3389/fpubh.2021.797267

**Published:** 2022-02-03

**Authors:** Ana Isabel López García, Florian Scharpf, Anke Hoeffler, Tobias Hecker

**Affiliations:** ^1^Development Research Group, University of Konstanz, Konstanz, Germany; ^2^Department of Psychology, Institute of Interdisciplinary Research on Conflict and Violence, Bielefeld University, Bielefeld, Germany

**Keywords:** school violence, teacher violence, teachers, school-based intervention, students, primary school

## Abstract

**Context:**

Although teacher violence at schools is a serious problem in Haiti, there is a lack of systematic evidence on the effectiveness of school-based interventions in reducing teacher violence in this low-income country.

**Objective:**

To test the effectiveness of the preventative intervention *Interaction Competencies with Children for Teachers (ICC-T)* aiming to reduce teachers' use of violent disciplinary strategies and to improve their interaction competences with children in the Haitian context.

**Design, Setting, Participants:**

The study is designed as a two-arm matched cluster randomized controlled trial. The sample consists of 468 teachers and 1,008 children from 36 (community and public) primary schools around Cap-Haïtien (Département du Nord) in Haiti. Data will be collected in three phases, before the intervention, and 6 and 18 months after.

**Intervention:**

In the group of intervention schools, ICC-T will be delivered as a 5-day training workshop. Workshop sessions are divided into five modules: 1) improving teacher-student interactions, 2) maltreatment prevention, 3) effective discipline strategies, 4) identifying and supporting burdened students, and 5) implementation in everyday school life.

**Main Outcome Measure:**

The main outcome measure is teacher violence assessed in two ways: (i) teachers' self-reported use of violence, and (ii) children's self-reported experiences of violence by teachers.

**Conclusions:**

Prior evaluations of ICC-T had been conducted in sub-Saharan Africa with promising results. This study will test for the first time the effectiveness of this intervention outside the context of sub-Saharan Africa.

## Introduction

Corporal punishment is still a widespread phenomenon in schools worldwide, particularly in low-income countries (1). This practice consists of the use of physical force inflicted by teachers with the purpose of correcting a child's behavior and may include beatings with the use of hands or objects, such as a cane or stick, shaking, pinching or kicking students or forcing them to adopt painful bodily postures for a long time (2, 3). Research from around the world shows that corporal punishment can have negative and long-lasting repercussions on children's physical and emotional well-being (4, 5). In schools, corporal punishment has been related to decreased school performance and higher dropout rates (2). Despite having received less empirical attention, emotional violence by teachers, including practices such as belittling, ignoring and humiliating students, has also been related to increased levels of students‘ mental health problems and academic deficits (6–8). Through their effects at the individual level, disciplinary violence can hinder development at the country level (9). This is recognized in the United Nations' (UN) Development Agenda 2030, aiming to end all forms of violence against children as one of the UN's Sustainable Development Goals (SDG 16.2). Assessing the magnitude of teacher violence and devising effective prevention strategies is therefore an important step to promote development in low-income countries. The country of Haiti, ranking at 170 out of 189 countries in the Human Development Index, provides an appropriate setting to investigate the effects of reducing violence at school on children's well-being and psychosocial functioning.

Although Haiti has made significant progress in both getting children into school and addressing teacher absenteeism in recent years, school performance remains very poor^1^. Among the many obstacles that Haitian children face is violent discipline, despite increased efforts of the country to protect children in the past decades. In 1995, Haiti signed the Convention on the Rights of the Child, which protects children against all forms of violence (Art. 19). Corporal punishment of children at both home and schools is explicitly forbidden by the Law Against Corporal Punishment of Children (2001) (10). Accordingly, any person or authority who violates the physical integrity of a child will be dismissed and prosecuted under the Penal Code. If the offender is an institution—such as a school—its premises shall be closed. This law demands schools to establish a code of conduct to monitor and sanction offenders.

However, corporal punishment is still regularly practiced at both home and schools. The 2012 Violence Against Children (VAC) survey reveals that 67% of Haitian adolescents and young adults aged between 13 and 24 have experienced physical violence during childhood by a family member/caregiver or public authority figure (11). Of these, the most common perpetrators of disciplinary violence were mothers (47%) and fathers (40%), followed by aunts and uncles. Among public authority figures, the primary perpetrators are teachers (11). In an unpublished pilot feasibility study of the proposed intervention, Haitian primary school teachers named yelling at and insulting students, caning them, forcing them to kneel down or standing at the wall for a period of time as well as expelling them from the classroom as most common discipline methods at their schools.

Research suggests that those who extensively use corporal punishment as a disciplinary strategy do not tend to perceive it as a form of physical abuse, may be unaware of the negative consequences of this violence, and often lack the skills necessary to manage children's misbehavior in a non-violent way (12). In Haiti, nearly one-third of household heads believe that violence is necessary to educate children^2^. A study conducted in 39 primary schools in Haiti's Nord-Est department found that school principals view violence as a necessary tool to maintain discipline (13). Moreover, media accounts show that most parents in Haiti regard schools using disciplinary violence as rigorous and serious institutions^3^.

Another reason why corporal punishment at schools is so prevalent has to do with the lack of capacity of the Haitian state to train and monitor schools and teachers (14). Haiti has suffered a devastating earthquake in 2010, and more recently one in 2021. This is important since evidence shows that violence against children can intensify after disaster settings and situations of unrest or conflict (15–17). Eleven years after the earthquake, the need for schooling is still dire. Presently, there are no rules (at least not enforced) for opening a school or becoming a teacher in Haiti. In Haiti, 80% of primary schools are non-state schools (and three quarter of them operate without a license of the Ministry of Education)^4^. Furthermore, only 20% of primary school teachers receive formal basic pedagogical training^5^.

Although school violence has been banned in Haiti, teachers lack training in alternative disciplinary strategies. A survey of 30 teachers in four primary schools in the capital Port-au-Prince showed that most of them rejected the use of violence. They resort to it mainly because violence is allowed or encouraged at schools (18). Media reports also confirm that teachers resort to violence upon the instructions of school principals and/or the consent of parents^6^. Providing alternatives and changing beliefs around violent discipline in schools can thus be an important step toward development in this country.

A few interventions focusing on cultivating teachers' use of non-violent disciplinary strategies have shown to be effective in reducing the prevalence of corporal punishment at primary schools in low- and middle-income countries, including the Good School Kit in Uganda (9) and the Irie Classroom Toolbox in Jamaica (19). Currently, there is only one evaluated intervention focusing both on changing attitudes toward violence and on providing non-violent disciplinary methods, namely *Interaction Competencies with Children—for Teachers* (ICC-T) (3, 20, 21). Although violent discipline at schools is a serious problem in Haiti, we lack systematic evidence on the effectiveness of school-based interventions in reducing teacher violence in this low-income country.

The purpose of this study is to evaluate the effectiveness of ICC-T in reducing teacher violence in Haitian primary schools through a two-arm matched cluster-randomized controlled trial (CRCT). In this study, violence by teachers refers to acts of both physical and emotional violence. Prior CRCTs of this intervention had already been undertaken in Tanzania and Uganda (3, 20, 21). Results suggest that ICC-T is a successful strategy to reduce violence by teachers and improve teacher-child relationships. While promising, the effectiveness of ICC-T in other low-income countries outside sub-Saharan Africa has not been tested yet. The proposed study will thus be the first CRCT testing ICC-T in the Western Hemisphere.

## Methods and Analysis

### Study Design and Sampling

**Figure 1:**
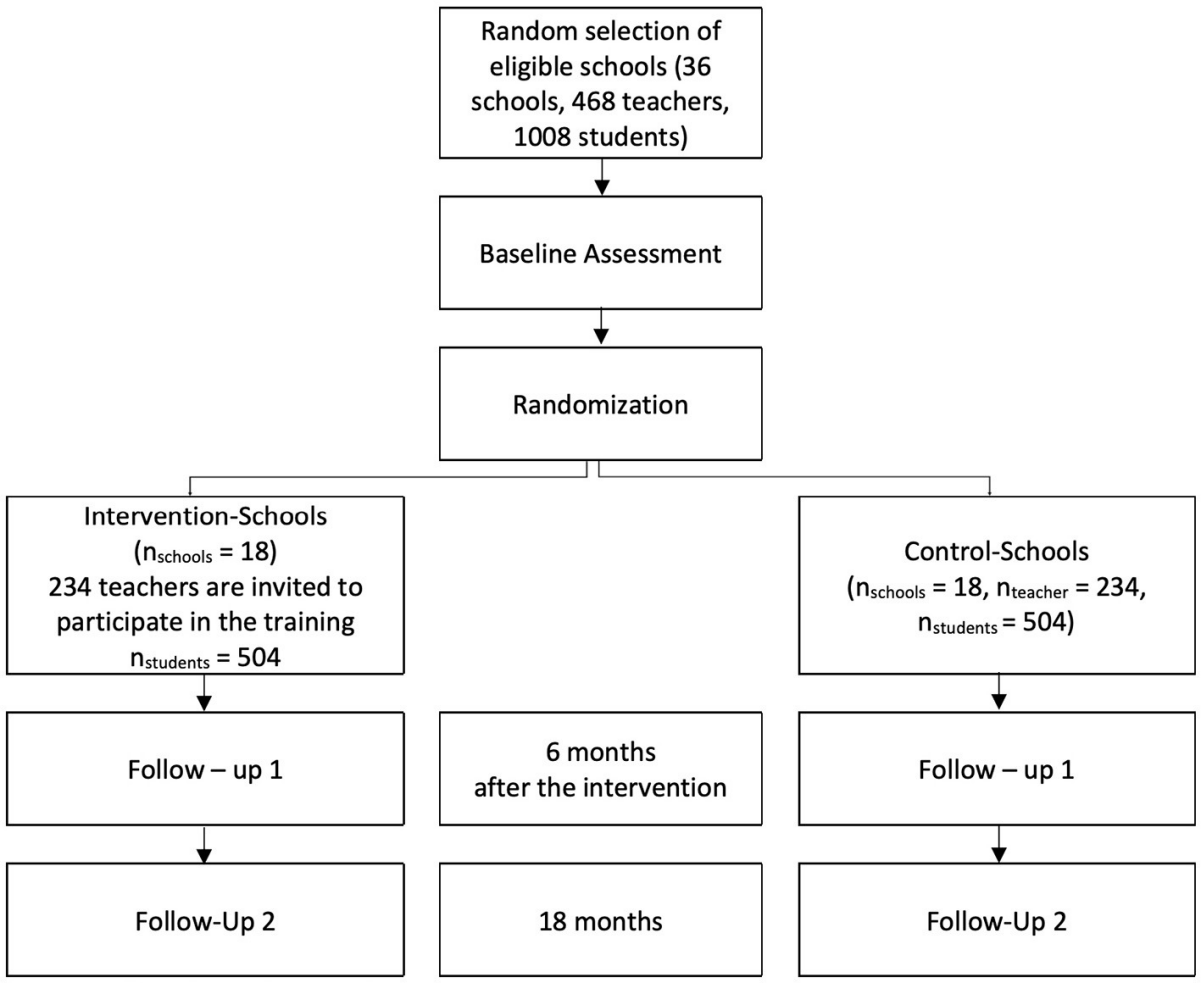
Flow chart of the study design.

**Figure 2:**
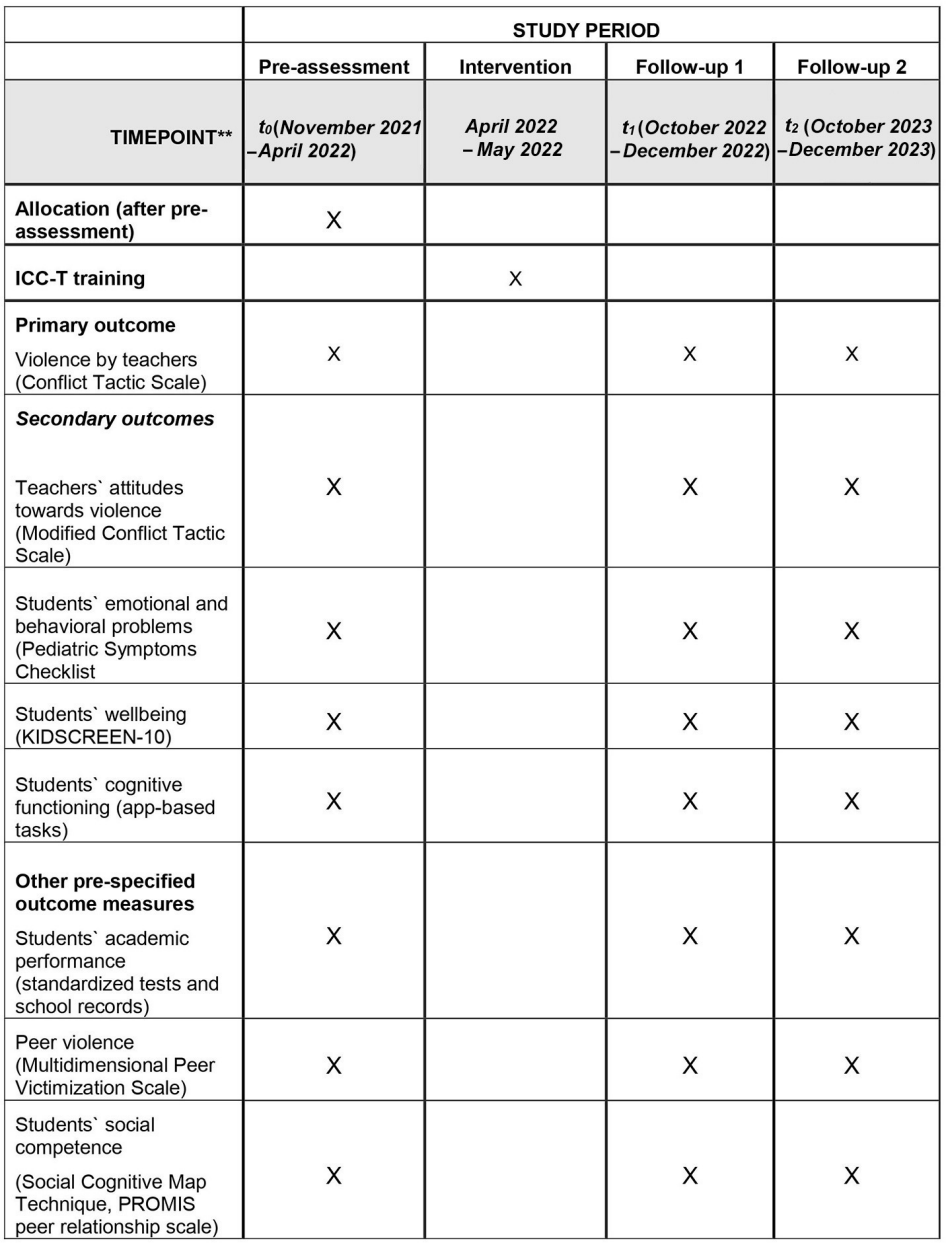
Study timeline.

The study is designed as a two-arm matched cluster-randomized controlled trial. It will be based on (at least) 36 primary schools from six of the overall 19 communes in the *Départment du Nord* in Haiti. For logistical reasons, this study will focus on the six communes in which all schools are reachable within 2 h drive from *Cap-Haïtien*, the capital of the *Départment du Nord: Cap-Haïtien, Quartier-Morin, Limonade, Acul-du-Nord, Plaine-du-Nord* and *Milot*. As there are only few schools in the commune *Quartier-Morin*, the schools in this commune will be combined with those schools in the two neighboring communes *Dondon* and *Grande-Rivière-du-Nord* that are reachable from *Cap-Haïtien* within 2 h. Three data collection phases will be undertaken: a baseline (pre-) assessment before the intervention (t_0_), a first follow-up assessment ~6 months after the intervention (t_1_) and a second follow-up 18 months after the intervention (t_2_). The study flowchart is displayed in Figure 1, while the timeline is shown in Figure 2. The estimated total costs of the project amount to US $ 220,000.

### Power Calculation

We conducted an *a priori* power analyses to determine the number of clusters (schools) for the trial as well as the number of participants (students and teachers) per cluster. Following a previous trial of ICC-T at Tanzanian secondary schools (21), we expected moderate (δ = 0.25) and large effects (δ = 0.35) on student-reported and teacher-reported violence by teachers, respectively. Based on previous and ongoing trials of ICC-T (3, 20, 21), we expected an intra-class correlation coefficient (ICC) of 0.03 and 0.05 for student- and teacher-reported violence, respectively. Moreover, the covariance between baseline and follow-up scores of violence at the school level was *r* = 0.30. Given all specified parameters (α = 0.05; power = 0.80; effect size students: δ = 0.25, effect size teachers: δ = 0.35; ICC students = 0.03, ICC teacher = 0.03; *R* = 0.30), no drop out at the school-level and an estimated dropout rate of 30% for teachers and 25% for students, in total 36 schools with 28 students and 13 teachers per school are required to detect the expected effects. The total sample size is therefore at least *n* = 1,008 children and *n* = 468 teachers.

### Schools

Selected schools must meet the following inclusion criteria: (1) teachers should not have received any training on violence prevention in the past; (2) the school should be a community or public mixed-gender school; (3) the school should have at least 13 teachers and at least 40 children in the 4th class of primary school (see power calculation above). In case of fewer than 13 teachers or fewer than 40 students in the relevant grade, the selected school will be combined with a neighboring school (within 15 km) into a school cluster. Schools that meet these criteria will be listed in alphabetical order and a random selection of six schools/school clusters in each of the six communes of Haiti's Département du Nord will be executed using http://www.random.org. In each commune, three schools or school clusters will be allocated to the treatment group (i.e., teachers will receive the ICC-T sessions), and the other three schools or school clusters into the control group (i.e., teachers will not receive the ICC-T sessions) after baseline assessment. All random selections and allocations will be performed by independent research staff.

### Participants

All children enrolled in grade 4, i.e., about 9 to 11 years of age, are eligible for participation. This grade is selected because of children‘s ability to comprehend the questionnaire items and their availability during follow-up assessment (children should not have made the transition to secondary school before the 2nd follow-up assessment is completed). To account for a lower response rate, 40 students in grade 4 will be stratified by gender and then randomly selected. Although our target sample is of at least 13 teachers per school, all teachers in the selected schools will be included in the study sample. Both children and teachers will be assessed at pre-assessment (baseline) and the two follow-up assessments, 6 months, and 18 months after the interventions.

### Assessment Procedures

As part of the recruitment process, the implementing partner—P4H Global, a Haitian NGO providing training programs to teachers in Haiti—will visit the selected 36 schools to advertise the study, meeting first with the school principals. These visits serve to check the inclusion criteria for schools and to ensure the willingness of principals to participate in the study.

The local partner will shortly return to schools to register and enroll the teachers and the children. To ensure common and clear understanding of the relevant details of the study, the local partner will provide information to selected students and voluntarily participating teachers in a formal information session. Parental informed consent will be sought for each selected student through information sessions for parents at the school or through letters sent out to parents. After baseline, schools will be allocated into treatment and control groups, and the program will be delivered to those in the treatment group after the baseline (pre-) assessment is completed.

Data will be collected from both control and treatment schools. Both teachers and children will be surveyed. Data collection will take place in three stages: (i) t_0_, at baseline – before the intervention; t_1_, 6 months after the intervention; and t_2_, 18 months after the intervention. Each data collection phase will take approximately two and a half months. Data will be collected using tablet-based surveys and the software Survey-to-go (22). Since floor effects were detected in the feasibility study, surveys will not be self-administered but conducted as interviews in Haitian Creole. A team of enumerators (external to the local partner facilitators so that results are not contaminated) will be trained in the use of this survey methodology and the collection of data. The local partner leadership will recruit and supervise the enumerators, who will be trained prior to the implementation of the program by the research team. Enumerators should be native Haitians, fluent in both English and Haitian Creole and ideally have a background in psychology, pedagogy, education, or related disciplines. The local partner and the research team will provide onsite supervision structure to this team of enumerators through the duration of the study. Enumerators will be blind regarding the allocation of the schools.

Children whose parents provided informed consent and who themselves consented to participate will be interviewed by the enumerators within the school premises. To guarantee privacy, the children will be seated in such a way that no one can hear how they answer to the questions. Teachers who agree to participate and provide informed consent will be interviewed during breaks between school lessons (or after work at school). When acquiring participants' informed consent, enumerators emphasize that participants can withdraw from the study at any time without any negative consequences and that, if they decide to do so, they can object to the use of their previously collected data. Interviews will take (on average) 45 min for teachers and 60 min for children at baseline and at the follow-up assessments. In addition, children will participate in a standardized numeracy and literacy testing and a cognitive assessment at each time point.

### Intervention Description

In the treatment condition, ICC-T will be delivered as a 5-day training workshop (9 h per day). Drawing on attachment, behavioral and social learning theories, ICC-T provides teachers with practical information and tools to prevent the use of violent disciplinary strategies in class and to improve their interaction competencies with children. Workshop sessions are divided into five modules (12): (1) improving teacher-student interactions, (2) maltreatment prevention, (3) effective discipline strategies, (4) identifying and supporting burdened students, and (5) implementation of ICC-T components in everyday school life. Sessions about *teacher-student interactions* aim to increase teachers' understanding of children's behavior, raise awareness of their responsibility of being a role model for students and foster positive teacher-student communication and interaction. Sessions on *maltreatment prevention* aim to raise awareness of the negative consequences of violent discipline for children's well-being. Teachers are encouraged to reflect on their own experiences of violent discipline as a child and to connect their own experiences and feelings to their current discipling behavior and its consequences. Sessions on *effective discipline strategies* aim to equip teachers with practical tools on how to maintain and reinforce desired behaviors of students and how to change misbehavior. The sessions on *identifying and supporting burdened students* aim to raise teachers' awareness of common internalizing and externalizing problems of children and to provide strategies how to support children with these problems in the school context. Finally, in the sessions on *implementation* teachers are encouraged to devise ways of how they can apply the ICC-T components in everyday school life and to create long-term support strategies to promote the sustainability of the intervention content.

Throughout the ICC-T sessions, teachers are invited to participate actively and think about how they can implement the workshop's components and acquired skills in their daily work. To create a trusting and open environment, participants are encouraged to discuss openly about their work problems, needs, and prior experiences with violent discipline. To promote sustainability, the workshop includes exercises for practicing, reinforcing, and repeating the workshop's content, activities that promote teachers' self-reflection about their own behavior, team-building measures as well as the organization of a peer consulting system in schools.

### Intervention Procedures

Each ICC-T training workshop will be delivered by two main facilitators with the assistance of one assistant facilitator. All facilitators are trained Haitian teachers, education specialists, and psychologists. They had already been certified by the research team as ICC-T facilitators. They will receive a series of refreshing training sessions from the research team before the delivery of the ICC-T to treatment-condition schools. All the materials for conducting the ICC-T sessions will be available in Haitian Creole, similarly all the presentations and discussion meetings during the workshop will be held in this language.

The ICC-T training workshop will be scheduled by our local partner, P4H Global, according to the current school calendar so that teachers have time to attend the 5 days of the workshop. All teachers who are employed at the treatment schools will be eligible to participate in the training. Participation in the training will be voluntary and free of charge. The local partner will provide food and beverages to participants as well as transport compensation (6 USD per person). Prior to their participation in the training, teachers will be given introductory (consent) forms, informing them of the voluntary nature of their participation as well as their right to withdraw from the training at any point. Teachers who have agreed to participate will sign the training informed consent form. The research team will ensure that the information shared during the workshop sessions remains confidential. No personal information will be disclosed to external sources.

Of the at least 36 schools that will participate in the study, the local partner will deliver the ICC-T program to half of the schools (at least 18). Workshop sessions will take place in 2 rounds. In the first round, at least 10 schools (about 130 teachers) will be trained. In the second round, at least 8 schools (about 104 teachers) will be trained. Each round of trainings will consist of five ICC-T trainings taking place in different locations. A total of 9 workshops (2–3 schools per workshop, maximum 30 participants per workshop) will be delivered by the local partner's trained staff. As one workshop takes 5 days and multiple workshops can be delivered by the local partner at the same time, all workshops can be delivered within a time window of about 1 month.

Treatment fidelity will be monitored in several ways. First, both facilitators will fill out a short purpose-built questionnaire after each session including items on the session's duration, applied methods, and perceived uptake of the session content by participants as well as a checklist on possible deviations from the intervention manual and didactical aspects. Second, at the end of each workshop day, four randomly selected participants will be asked to fill out a purpose-built questionnaire on their perceived understanding of that day's training content and the helpfulness of the applied methods in delivering the content. Third, all participants will be asked to evaluate the training contents and methods using a purpose-built questionnaire at the end of the workshop. Fourth, two independent raters will evaluate video and audio recordings of pre-determined sequences of ~10 min to determine whether intervention workshops were implemented in line with the manual.

### Control Schools

No ICC-T workshops will be delivered to teachers working at control-condition schools. That said, the local partner will have close contact with control schools to ensure that teachers do not receive any training of a similar nature during the total study duration. School (vice)principals should consent to this. Data collection at control schools will be conducted both at baseline (pre-assessment) and in two follow-up phases.

### Study Measures

Following established international guidelines, Haitian Creole native speakers will translate all the forms and measures from English into Creole and then back into English in a blind written form. The back-translated items will then be compared with the original items to verify that the translation has been correctly undertaken and the content is equivalent. To ensure high objectivity and reliability during data assessment, standardized procedures on questionnaire administration will be developed and implemented. All surveys will be undertaken in Haitian Creole. Prior to data collection, a pilot study in one primary school (which will not be included in the trial) will be conducted to ensure the feasibility of the questionnaire administration.

The main outcome measure is teachers' use of violence. This will be measured in two ways: (i) teachers' self-reported use of violence, and (ii) children's self-reported experiences of violence. Secondary outcome measures are teachers' attitudes toward violent discipline, children's mental health problems and quality of life as well as their cognitive performance. Additionally, we will assess children's experiences of peer violence, social competence and their academic performance through a standardized literacy and numeracy test and children‘s scores in Mathematics, Creole-French, Science, Social science, and General Studies in the mid-term. These scores will be provided by the school administration.

### Children

#### Experience of Violence

At pre-assessment and at the follow-up assessments, children's exposure to violence by teachers in the past week will be assessed using an adapted version of the Conflict Tactic Scale (CTS) including 16 items on experienced physical violence, 7 items on experienced emotional violence and 3 items on witnessed violence by teachers. These items are scored on a 6-point Likert scale ranging from 0 (never) to 5 (more than 10 times). Subscale scores are derived by summing up all item scores. The original CTS has been used worldwide as a measure of physical and emotional maltreatment of children and its validity has been demonstrated by numerous studies (23). The CTS has also been used to assess students' self-reported experiences of violence by teachers in different cultural settings, e.g., in Tanzania (8) and Turkey (24). While the total score of physical and emotional violence had acceptable (α = 0.72) to excellent Cronbach's alpha coefficient (α = 0.88) in a previous study (24, 25), the two subscales of physical violence and emotional violence exhibited lower internal consistency (α = 0.55 and α = 0.60, respectively) in another study (8). The low reliability can be explained by the fact that the items measure rather rare events and that the correlation between items, which is the basis of alpha, is low due to extreme skewness (26).

#### Mental Health Problems

The Pediatric Symptom Checklist—Youth Report [PSC-Y; (27)] will be used to assess children‘s emotional and behavioral problems. The PSC-Y consists of 35 items rated on a 3-point Likert scale from 0 (never) to 2 (often), which can be summed up to a total score of emotional and behavioral problems ranging from 0 to 70. Factor-analyses of the parent- and youth-report version of the PSC revealed a 3-factor structure of internalizing problems, externalizing problems and attention problems (28, 29). In the original validation study, the PSC-Y demonstrated good concurrent validity with parent- and teacher-reports of children's emotional and behavioral problems as well as moderate test-retest-reliability (27). Adapted versions of the PSC haven been used in different cultural settings including Botswana (30), Uganda (31) and Turkey (32) with good psychometric properties, indicating the instrument‘s cross-cultural applicability. For instance, in a sample of children and adolescents in Botswana, the PSC-Y showed high internal consistency with a Cronbach's alpha of 0.89 and also good sensitivity and specificity to detect children with increased levels of mental health problems (30).

#### Quality of Life

The KIDSCREEN-10 (33) will be used to assess children‘s perceived quality of life. The KIDSCREEN-10 conceptualizes quality of life as a multidimensional construct covering physical, emotional, social and behavioral aspects of well-being and functioning. The 10 items referring to the past week are rated on a 5-point Likert scale from 0 (not at all) to 5 (extremely). The KIDSCREEN-10 has shown to be a valid (Cronbach's alpha of 0.82 and test-retest reliability of 0.70) and cross-cultural comparable tool to assess children's and adolescents' self-reported quality of life (33).

#### Cognitive Functioning

Four classical tasks implemented in the Android application Psych Lab 101 (34) will be used to assess different aspects of children's cognitive functioning: A visual search task (selective attention), a numerical stroop task (ability to resist interference by distracting information), a delayed match-to-sample task (working memory capacity) and a continuous performance task (impulsivity). These tasks were selected because they are independent of language and they cover “core” cognitive abilities that have been shown affected by exposure to maltreatment (35, 36). Prior to data collection, a pilot-assessment will be conducted to ensure feasibility of the tablet-based assessment.

#### Academic Performance

Children's academic performance will be measured using a standardized numeracy and literacy test. This test is based on standardized tests of numeracy and literacy skills developed by the Uwezo initiative (37), which have been applied in large-scale surveys in East Africa to assess learning outcomes of primary school children. The included tasks will focus on essential numeracy and literacy skills independent of national curriculum and will be evaluated by educational experts in terms of their applicability for the study context. In addition, we will request from the school administration children's scores in Mathematics, Haitian Creole, English, Science, Social Science, and General Studies in the mid-term exam as a direct indicator of school performance.

#### Social Competence

Children's social competence will be assessed in two ways. First, a well-established peer-nomination procedure, the social cognitive map (SCM) technique (38), will be applied to assess children‘s social status in their peer networks. This procedure asks children to name a group of children in their class to which they belong as well as other groups of friends in their class. Based on the number of nominations as members of a group, the social centrality status of individual children can be determined (39). Moreover, children are asked to nominate three classmates they like most and three they like least, which yields an indicator of social preference status for each child. The SCM technique makes it possible to reliably identify social groups with proportions of respondents from a social network as small as 50% (39). The technique has been successfully implemented in a previous study with primary school children in different cultural settings e.g., in Tanzania (40). Second, the 8-item short form of the PROMIS pediatric peer relationship scale (41) will be used to assess the quality of children‘s relationships with peers and friends through their self-report. The items are rated on a 5-point Likert scale from 0 (never) to 5 (almost always) and refer to the past 7 days. The scale has been used in various cultural settings including a sample of child patients in Malawi (42). Cronbach's alpha coefficients ranged between 0.71 (42) and 0.85 (43), indicating acceptable to good internal consistency.

#### Peer Violence

We will assess children's experiences of violence by peers using the 24-item version of the Multidimensional Peer Victimization Scale (MPVS) (44). Four items each assesses the six subtypes physical victimization, verbal victimization, social manipulation, attacks on property, electronic victimization and social rebuff. The original 16-item and the 24-item version of the MPVS have shown good psychometric quality with Cronbach's alpha coefficients ranging from 0.74 to 0.96 (44). We will additionally assess sexual victimization by peers using four items from the adolescent version of the Sexual Experiences Survey (45). Two items each will cover sexual harassment and sexual assault by peers.

### Teachers

#### Use of Violent Discipline

Teachers' use of physical and emotional violence against students in the past week will be assessed using the same modified version of the CTS as for children. Hence, the items (except for the three items on witnessed violence) and the answer scale are the same (see above). The CTS scale has proved useful in assessing teachers' self-reported use of violence in the classroom across various cultural settings (24, 25, 46). Cronbach alpha coefficients ranged between 0.73 (46) and 0.83 (24), indicating acceptable to good internal consistency.

#### Attitudes Toward Discipline

We will use an adaptation of the CTS to assess teacher's positive attitude toward the use of violent discipline (Nkuba et al., 2018). The items are the same as described before, but this time scored on a four-point scale Likert scale ranging from 0 (never OK) to 3 (always or almost always OK). The scores for each subscale are then summed up into one score for physical violence (ranging from 0 to 48) and one for emotional violence (ranging from 0 to 21). The measure showed good internal consistency (Cronbach's alpha of 0.84) in a previous study with teachers in Tanzania (46).

### Measures Against Bias

To minimize recruitment bias, a stratified random sampling approach is used. To minimize bias from using unvalidated outcome measures, a careful selection of the assessment instruments has been undertaken. Since the school allocation will be executed at the cluster level and by independent researchers, the data collectors will be blind to the treatment conditions of the schools. To further minimize the risk of contamination between treatment conditions, there will be no contact between the intervention facilitators and data collectors. Although the intervention participants will not be blind regarding whether they belong to the treatment or the control group, the use of violence by teachers will also be assessed through children's self-reported exposure to violence. To avoid incomplete accounting of participants and outcome events, the analysis will treat groups as randomized (“intention to treat”).

### Statistical Analysis

Pre-assessment data will be used to assess the prevalence of maltreatment and violence as well as children's mental health and well-being. A longitudinal analysis (including the follow-up assessments) will then be undertaken using an intent-to-treat approach. As drop-outs and missing data at follow-up assessment are likely given the longitudinal study design, we aim to apply full information maximum likelihood estimation to obtain unbiased parameter estimates.

Our main analysis of the primary and secondary outcomes will be time × group interaction effects using multilevel linear modeling. By randomly assigning schools to the intervention and control condition, we do not expect systematic differences between schools, teachers, and students in the two groups. However, we will examine differences between the groups in sociodemographic characteristics on the level of the school (e.g., material conditions), teachers (e.g., socioeconomic status, teaching experience) and students (e.g., socioeconomic status, gender). In case of significant differences, we will control for these differences in all analyses. Latent growth modeling or cross-lagged path models will be used to estimate the directional influence of violence by teachers on primary and secondary outcome variables over time. Results will be presented including appropriate effects sizes and with a measure of precision (95% confidence intervals). Effect sizes of η^2^ ≥ 0.01, η^2^ ≥ 0.06 and η^2^ ≥ 0.14 will be considered as small, moderate, and large effects, respectively (47).

## Discussion

Violence by teachers represents a considerable risk for children's well-being and development, which also entails tremendous direct and indirect costs for societies and thereby hinders their socio-economic progress. Although Haiti, the poorest country in the Western hemisphere, has officially abolished violent punishment of children at school, quantitative and qualitative evidence suggests that violence by teachers and school staff are still widespread in Haitian schools. Studies further point to the relevance of long-standing beliefs and attitudes about the effectiveness of violence to discipline children and a lack of adequate training in non-violent discipline strategies as factors contributing to teachers' ongoing use of violence. ICC-T has been developed as a training for teachers that aims to challenge and reduce attitudes favoring violence and to equip teachers with alternative non-violent discipline strategies. Prior studies provided initial evidence for the feasibility and effectiveness of ICC-T in reducing violence by teachers in Uganda and Tanzania (3, 12, 21). However, further studies that replicate these findings beyond the context of sub-Saharan Africa are needed.

The theory of change of the intervention is based on two pillars: On the one hand, teachers are encouraged to develop empathy with students, which is expected to initiate a change of attitudes toward the use of violence. On the other hand, teachers actively learn and practice non-violent discipline strategies, which counteracts the feelings of helplessness and loss of control that are often related to the use of violence. A variety of different didactic and interactive methods including self-reflections, discussions, role-plays, practical exercises, and theoretical input are used to facilitate the process of changing attitudes and cultivating non-violent action alternatives. Special emphasis is put on the sustainability of the intervention content to achieve a lasting impact in the school environment by encouraging teachers to devise ways how to integrate the newly learnt strategies into their daily work and by implementing peer support and consultation networks.

Haiti is an example that legal bans and international conventions may be necessary, but not sufficient to stop violence against children in the school setting (48). We argue that it is crucial to address attitudes and norms about the violent discipline of children as well and to work together directly with educators. A reduction of violence in the school setting has also shown to precede a wider societal change in attitudes and behaviors regarding corporal punishment in various countries (49). The study will be conducted against the backdrop of political turmoil in Haiti following the assassination of the president in July 2021 and amid a precarious humanitarian situation that has been aggravated by the earthquake in August and by the ongoing Covid-19 pandemic. As research indicates an increase of multiple risk factors for violence against children in the wake of political conflict, disasters and the pandemic (50), programs aiming to prevent violence against children in their most important social settings, i.e., at school and at home, are sorely needed.

Our primary outcome of interest is teachers' use of violence against children in the past week. As teachers' reports of violence may be biased in the same direction as the intervention effect, we also consider students' self-reports of violence by teachers. We expect the reduction of teachers' use of violence to be reflected in a change of the secondary outcome of teachers' attitudes toward violent discipline. The choice of secondary outcomes on the students' side is based on the assumption that a school environment characterized by less violence by teachers will have positive effects on students' mental health, quality of life and cognitive abilities. Moreover, we expect that a lower use of violence by teachers will also lead to less victimization by peers, as teachers are likely to act as non-violent role models for children. We assume that the long-follow up interval of 18 months will be enough time for these effects to unfold. As the use and experience of violence are sensitive topics, we expect socially desirable responses to be a relevant issue in spite of societal norms in favor of violent discipline. Therefore, adequate training of enumerators in recognizing and dealing with social desirability is crucial. We opted for structured interviews as assessment method as this will allow enumerators to get more valid and accurate answers on the topics of interest.

### Limitations

The research team will ensure that individual participation is entirely voluntary and that any participant who would like to drop out the study at any stage can do so. This could however affect the findings, due to the longitudinal and experimental nature of this study. For instance, some eligible participants might refuse to participate in the study, or there could be potential dropouts throughout the project. Attrition among participating children and teachers may occur because of several reasons, such as teachers changing jobs, moving from one school to another, or truancy and absenteeism. Another challenge owes to the rootedness of attitudes toward the use of violence in culture and society as these beliefs might take time to be eradicated. Hence, the expected changes in attitudes and behavior should be considered as preliminary only. Finally, the generalizability of the study results can be affected, if only a few schools participate in the study.

## Ethics Statement

The study has been reviewed and approved by Institutional Review Board of the University of Konstanz. Written informed consent to participate in this study will be provided by the participating teachers themselves and the participants' legal guardian/next of kin of the participating children.

## Author Contributions

TH, ALG, and FS designed the study and drafted the manuscript. AH made significant contributions to the study design. All authors have read and approved the final manuscript.

## Funding

This study is funded by the Alexander von Humboldt Foundation. The funding body has no role in the design of the study and in writing the manuscript. We acknowledge the financial support of the German Research Foundation (DFG) and the Open Access Publication Fund of Bielefeld University for the article processing charge.

## Conflict of Interest

The authors declare that the research was conducted in the absence of any commercial or financial relationships that could be construed as a potential conflict of interest.

## Publisher's Note

All claims expressed in this article are solely those of the authors and do not necessarily represent those of their affiliated organizations, or those of the publisher, the editors and the reviewers. Any product that may be evaluated in this article, or claim that may be made by its manufacturer, is not guaranteed or endorsed by the publisher.

## Footnotes

^1^Ministry of National Education and Vocational Training (2015-2016). Résultats du recensement scolaire 2015-2016. https://fne.gouv.ht/wp-content/uploads/2020/02/rapport_recensement_scolaire00_25_05_17_vf.pdf.

^2^Demographic and Health Surveys (2016). Enquête Mortalité, Morbidité et Utilisation des Services EMMUS-V / EMMUS VI. Ministère de la Santé Publique et de la Population. https://www.mspp.gouv.ht/site/downloads/EMMUS%20V%20web.pdf.

^3^HPN Societe andCulture (2013). “Haïti-Éducation: Les châtiments corporels à l'école, l'autre problème du système”. HPN Societe & Culture. http://www.hpnhaiti.com/site/index.php/societe/9688-haiti-education-les-chatiments-corporels-a-lecoles-lautre-probleme-du-systeme.

^4^United Nations Development Programme (2014). “Millenium Development Goals – Country Report Haiti 2013.” United Nations. http://www.latinamerica.undp.org/content/rblac/en/home/library/mdg/HaitiMDGReport2013.html.

^5^Haiti's Ministry of National Education and Vocational Training (2017). “Politique nationale de formation des Personnels d'enseignement et d'encadrement (PNF/PEE) Plan de mise en œuvre” République d'Haïti. http://50.21.183.214/assets/strategiepays/412.pdf.

^6^Louis (2019). Fessée, Rigwaz et matinèt… que dit la loi en Haïtiè Ayibopost. https://ayibopost.com/fessee-rigwaz-et-matinet-que-dit-la-loi-en-haiti/.
